# Altering the N-terminal arms of the polymerase manager protein UmuD modulates protein interactions

**DOI:** 10.1371/journal.pone.0173388

**Published:** 2017-03-08

**Authors:** David A. Murison, Jaylene N. Ollivierre, Qiuying Huang, David E. Budil, Penny J. Beuning

**Affiliations:** Department of Chemistry and Chemical Biology, Northeastern University, Boston, MA, United States of America; Indian Institute of Science, INDIA

## Abstract

*Escherichia coli* cells that are exposed to DNA damaging agents invoke the SOS response that involves expression of the *umuD* gene products, along with more than 50 other genes. Full-length UmuD is expressed as a 139-amino-acid protein, which eventually cleaves its N-terminal 24 amino acids to form UmuD′. The N-terminal arms of UmuD are dynamic and contain recognition sites for multiple partner proteins. Cleavage of UmuD to UmuD′ dramatically affects the function of the protein and activates UmuC for translesion synthesis (TLS) by forming DNA Polymerase V. To probe the roles of the N-terminal arms in the cellular functions of the *umuD* gene products, we constructed additional N-terminal truncated versions of UmuD: UmuD 8 (UmuD Δ1–7) and UmuD 18 (UmuD Δ1–17). We found that the loss of just the N-terminal seven (7) amino acids of UmuD results in changes in conformation of the N-terminal arms, as determined by electron paramagnetic resonance spectroscopy with site-directed spin labeling. UmuD 8 is cleaved as efficiently as full-length UmuD *in vitro* and *in vivo*, but expression of a plasmid-borne non-cleavable variant of UmuD 8 causes hypersensitivity to UV irradiation, which we determined is the result of a copy-number effect. UmuD 18 does not cleave to form UmuDʹ, but confers resistance to UV radiation. Moreover, removal of the N-terminal seven residues of UmuD maintained its interactions with the alpha polymerase subunit of DNA polymerase III as well as its ability to disrupt interactions between alpha and the beta processivity clamp, whereas deletion of the N-terminal 17 residues resulted in decreases in binding to alpha and in the ability to disrupt the alpha-beta interaction. We find that UmuD 8 mimics full-length UmuD in many respects, whereas UmuD 18 lacks a number of functions characteristic of UmuD.

## Introduction

*Escherichia coli* (*E*. *coli*) cells that are exposed to exogenous or endogenous DNA damaging agents invoke the SOS response that involves the induction of at least 57 genes [1, 2]. The SOS response is temporally divided into two phases: the initial phase that allows time for error-free pathways to act, and a potentially mutagenic damage tolerance phase that may ensure survival [1, 3]. Key participants in the later stage of the damage response, which is often referred to as SOS mutagenesis, include the *umuDC* and *dinB* gene products.

Full-length UmuD is a homodimer of 139-amino acid subunits, and is expressed 20–30 minutes after the induction of the SOS response [1, 3, 4]. UmuD interacts with the RecA:ssDNA nucleoprotein filament to facilitate the autocatalytic cleavage of the N-terminal 24-amino acids, forming UmuD′ [5–7]. The UmuD′ cleavage product is a homodimer of 115-amino acid subunits, and together with UmuC, forms the Y-family polymerase DNA pol V (UmuD′_2_C). This specialized DNA polymerase copies damaged DNA, albeit in a potentially error-prone fashion, in a process known as translesion DNA synthesis (TLS) [1, 4, 8, 9].

The N-terminal arms of UmuD are quite dynamic and can adopt multiple conformations, which regulate interactions with partner proteins [10–14]. UmuD can cleave in the *trans* (intermolecular) conformation, in which the arm of one monomer loops over and is cleaved by the active site of the adjacent monomer [11, 15]. Isoenergetic models of the UmuD dimer also suggest that the *cis* (intramolecular) conformation of the arms, in which each arm binds and is cleaved by its respective C-terminal domain, is possible [11]. The monomeric variant UmuD N41D also cleaves efficiently, which suggests that the *cis* conformation is likely an active conformation [16]. Additionally, the arms of UmuD may be bound (“arms down”) or unbound (“arms up”) from the C-terminal domain, which may significantly alter the interacting surface that is presented for binding [10, 11].

The *umuD* gene products interact with multiple factors involved in DNA replication and the SOS damage response [17]. UmuD and UmuD′ interact specifically with Y-family polymerases UmuC and DinB [1, 8, 9, 18]. The noncatalytic UmuDC complex protects cells from the potentially harmful effects of error-prone DNA replication by delaying SOS mutagenesis [3, 19]. This function is distinct from the role of UmuD′_2_C in error-prone TLS [1, 4, 8]. Additionally, both UmuD and UmuD′ interact differentially with the α polymerase, β processivity, and ε proofreading subunits of the replicative polymerase DNA pol III [20–22].

The *umuD* gene products are regulated at the transcriptional and post-translational levels. The *umu* operon is repressed by LexA and is one of the most tightly controlled in the SOS regulon [1]. Cleavage of UmuD to UmuD′ activates UmuC for TLS, and also removes the degradation signal for Lon protease [23]. UmuD and UmuD′ exist by themselves as homodimers, but can also exchange subunits to form the UmuDD′ heterodimer preferentially [11, 14, 24–26]. Both the UmuD′ subunit of the heterodimer and one full-length UmuD subunit of the UmuD homodimer are targeted for degradation by the ClpXP protease as a way of attenuating mutagenesis [23, 27, 28]. The N-terminal arm of UmuD harbors the ClpX recognition sequence, and thus UmuD acts as the delivery factor for its bound UmuD′ or UmuD partner [27, 28].

We previously showed that, even in full-length UmuD, the N-terminal arms are only loosely bound to the globular domain [10, 13]. In order to probe this further, in this work we constructed and characterized variants of UmuD possessing N-terminal truncations. These truncated proteins, UmuD 8 (UmuD Δ1–7) and UmuD 18 (UmuD Δ1–17), were used to study the conformation of the N-terminal arms, their effects on cleavage, and other cellular functions of the *umuD* gene products, as well as their effects on protein-protein interactions. We found that the loss of just the N-terminal seven amino acids of UmuD results in changes in conformation of the N-terminal arms, but this truncated UmuD maintains interactions with the α polymerase subunit of DNA polymerase III. Although UmuD 8 is cleaved as efficiently as full-length UmuD *in vitro* and *in vivo*, UmuD 18 is not cleaved to form UmuD′. We have also determined that UmuD 8 is proficient for UV mutagenesis, but intriguingly, sensitizes cells to UV radiation.

## Materials and methods

### Strains, plasmids, and proteins

Strains and plasmids used in this work are listed in Table 1.

**Table 1:** Strains and Plasmids.

Strains and Plasmids	Relevant Genotype	Source or Reference
***Strain***		
AB1157	*argE3*	Laboratory Stock
GW8017	AB1157 Δ*umuDC*	[29]
PB103	AB1157 Δ*umuDC* Δ*recJ*	P1 (JW2860)→ GW8017 [24, 30]
BL21 DE3		Laboratory Stock
***Plasmid***		
pGY9739	*o*^*c*^_*1*_ *umuD′C*; pSC101-derived, Spec^R^	[31]
pGB2	Vector; pSC101-derived, Spec^R^	[32]
pSG4	*umuD′*, Amp^R^	[33]
pSG5	*umuD*, Amp^R^	[33]

For protein expression and purification, NdeI restriction sites were introduced into the pSG5 expression vector [33] at positions 8 for UmuD 8 and 18 for UmuD 18 using a QuikChange kit (Agilent). There was already an NdeI site at the beginning of the *umuD* gene. The resulting plasmids were digested using NdeI (NEB), and re-ligated using T4 DNA Ligase (NEB). Mutations were confirmed by DNA sequence analysis (Massachusetts General Hospital Core Facility, Cambridge, MA). Mutagenic primer sequences are as follows:

UmuDAsp8NdeI2 forward (5′-GTTGTTTATCAAGCATATGGATCTCCGCG),

UmuDPhe18NdeI2 forward (5′-GTGACTTTTCATATGTTTAGCGATCTTGTTCAGTG), and their respective reverse complementary sequences. UmuD 8 and UmuD 18 were constructed in pSG5, and expressed and purified as previously described [33, 34]. In general, and unless otherwise noted, the biochemical experiments reported here used non-cleavable S60A variants to avoid complications due to the possibility of spontaneous cleavage.

For bacterial experiments, KpnI restriction sites were introduced into pGY9739 or the S60A derivative at positions 1 and 8, and 1 and 18, to create the UmuD 8 and UmuD 18 truncations, respectively, using a QuikChange kit (Agilent). The resulting plasmids were digested using KpnI (NEB), and re-ligated using T4 DNA Ligase (NEB). Mutations were confirmed by DNA sequence analysis (Massachusetts General Hospital Core Facility, Cambridge, MA). Mutagenic primers are as follows:

UmuDMet1KpnI19739 forward (5′-CTTCAGGCAGGGTACCATGTTGTTTATCAAGCCTG),

D_9739Asp8KpnI forward (5′-GTTGTTTATCGGTACCATGGATCTCCGCGAAATTGTGAC), UmuDPhe18KpnI2 forward (5′-CGCGAAATTGTGACTGGTACCATGTTTAGCGATCTTGTTC), and their respective reverse complementary sequences.

### *In vitro* characterization of truncated UmuD variants

Thermal shift assays of full-length UmuD, UmuD′, UmuD 8 and UmuD 18 were completed as previously described using a Bio-Rad CFX 96 Real-Time system [16]. Cross-linking of the UmuD N-terminal arms with 10 mM bis(maleimido)hexane (BMH, Thermo) at 40 μM protein was completed as previously described [11, 35], except proteins were visualized by Coomassie-stained SDS-PAGE. The RecA:ssDNA-dependent and alkaline cleavage assays were also carried out as previously described [16, 33].

Site-directed spin-labeling of purified UmuD variants for electron paramagnetic resonance experiments were carried out using the thiol-reactive nitroxide derivative, 3-iodomethyl-1-oxy-2,2,5,5-tetramethylpyrroline (Toronto Research Chemicals). Labeling chemistry was carried out as previously described [10]. Continuous wave experiments were performed at room temperature using a Bruker EMX instrument equipped with a high-sensitivity cylindrical cavity. Spectra were obtained using a 9.37 GHz microwave frequency, 6.0 mW microwave power, and 1.0 G 100 kHz field modulation amplitude. Spectra were aligned and scaled using MatLab (MathWorks) to illustrate differences in line shape as a function of nitroxide probe motion.

### UV survival and mutagenesis assays and inhibition of homologous recombination

Survival and mutagenesis assays were performed as previously described [33, 34]. Genetic transduction was carried out as previously described using P1*vir* Δ*yeaB* (Kan^r^) [36]. Values represent the average of at least three trials, and the error bars show the standard deviation.

### Immunoblotting

Immunoblotting procedure was completed as previously described [16], with rabbit polyclonal anti-UmuD/UmuDʹ antibodies [11]. Band densities were determined using ImageQuantTL software (GE).

### UmuD and DNA polymerase III α subunit binding by tryptophan fluorescence assay and FRET

Equilibrium dissociation binding constants *K*_d_ for the interaction between UmuD proteins and DNA polymerase III α subunit (pol III α) were determined by tryptophan fluorescence as previously described [21, 33]. Pol III α truncations α1–280 and α917–1160 were described previously [21] and were used here to localize UmuD 8 and UmuD 18 binding sites on α. UmuD was titrated into a solution of α.

Protein labeling with Alexa488 and Alexa647 (Life Technologies) and FRET assays were performed as previously described [21]. Purified UmuD variants were added to a final concentration of 40 μM, and incubated with fluorescent α and β proteins prior to analysis. FRET efficiency was calculated as previously described [21].

## Results

### Loss of N-terminal residues changes UmuD arm characteristics

**Fig 1:**
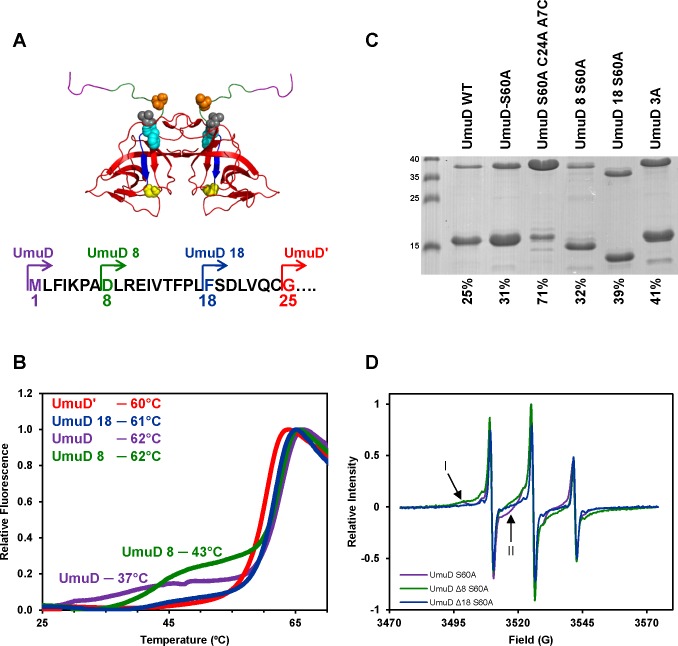
Characterization of UmuD 8 and UmuD 18 *in vitro*. (**A**) Model of UmuD (with arms down) showing residues 1–7 (purple), 8–17 (green) 18–24 (blue), 25–139 (red). UmuD 3A mutations T14 (orange), L17 (gray), F18 (cyan) and active site residue S60 (yellow) are also highlighted. (**B**) Thermal shift assays of UmuD proteins. Melting of the proteins as a function of temperature was monitored by changes in Sypro Orange (Life Technologies) fluorescence. Results for UmuD (purple), UmuD 8 (green), UmuD 18 (blue) and UmuDʹ (red) are shown using 40 μM protein. (**C**) UmuD protein arms were cross-linked using BMH. Percent of cross-linked dimers are indicated below the lanes. The cross-linking reaction was carried out for 10 min at room temperature after the addition of BMH. Protein was visualized by Coomassie stain. (**D**) Continuous wave EPR spectra of UmuD variants. Arrow I indicates line shape from a partially-immobilized species; arrow II shows line shape characteristic of elevated mobility.

In order to probe the dynamics and functions of the N-terminal arms of UmuD, we generated truncations lacking the N-terminal 7 or 17 residues, denoted as UmuD 8 and UmuD 18, respectively (Fig 1A). Truncating the N-terminal arms of UmuD to create UmuD 8 and UmuD 18 changed the melting profile relative to wild-type UmuD (Fig 1B). It was previously shown that wild-type UmuD melts in two transitions [13]. The transition at approximately 30 ˚C is attributed to release of the N-terminal arms from the globular C-terminal domain, and the second transition at approximately 60 ˚C is associated with melting of the globular domain [13]. The arms of UmuD′ (residues 25–40) are not in contact with the C-terminal domain; therefore, only one melting transition is observed at approximately 60 ˚C for UmuD′ [13]. The melting profile for UmuD 18 resembles that of UmuD′ with a single transition observed at 62 ˚C (Fig 1B). This suggests that the N-terminal arms of UmuD 18 are also dissociated from the C-terminal domain. Conversely, the melting profile of UmuD 8 resembles that of wild-type UmuD in which there is an initial melting transition at 43 ˚C for UmuD 8. This observation is consistent with a model in which the first transition is due to dissociation of the N-terminal arms from the globular domain. Thus, the arms of UmuD 8 are apparently of sufficient length to interact with the globular domain, whereas the arms of UmuD 18 are presumably too short to form a stable interaction.

The conformation of the N-terminal arms of UmuD 8 and UmuD 18 was also assessed by cross-linking with the homobifunctional cross-linker bis(maleimido)hexane (BMH). The BMH cross-linker is 13 Å in length and reacts with free cysteine thiols. The model of wild-type UmuD with N-terminal arms in the “down” conformation shows that the C24 residues within the dimer are separated by a distance of 20 Å (Fig 1A) [11]. Therefore, the arms must be unbound from the C-terminal domain for cross-linking of the single Cys residues to occur. UmuD S60A C24A A7C was used as a control to represent maximal cross-linking as the first few residues of the UmuD arm are highly dynamic [13]. As expected, the UmuD S60A C24A A7C variant was cross-linked the most readily (71%) due to the position of the cysteine near the end of the arm. UmuD 8 S60A (32%) and UmuD S60A (31%) exhibited similar cross-linking efficiencies, which were slightly lower than those of UmuD 18 S60A (39%) and UmuD 3A (41%) (Fig 1C). UmuDʹ was not used in this assay because full-length UmuD contains a single cysteine residue C24 which is at the cleavage site and is removed upon cleavage. The UmuD 3A variant possesses three alanine mutations (T14A L17A F18A) and is considered a UmuD′ mimic because the arms weakly interact with the C-terminal globular domain [11, 13]. Together, these results show that deletion of the first eight residues does not change the cross-linking efficiency compared to UmuD S60A, but deletion of the first 18 residues causes increased cross-linking efficiency, which is likely due to reduced interaction between the arms and the globular domain in the case of UmuD 18.

**Fig 2:**
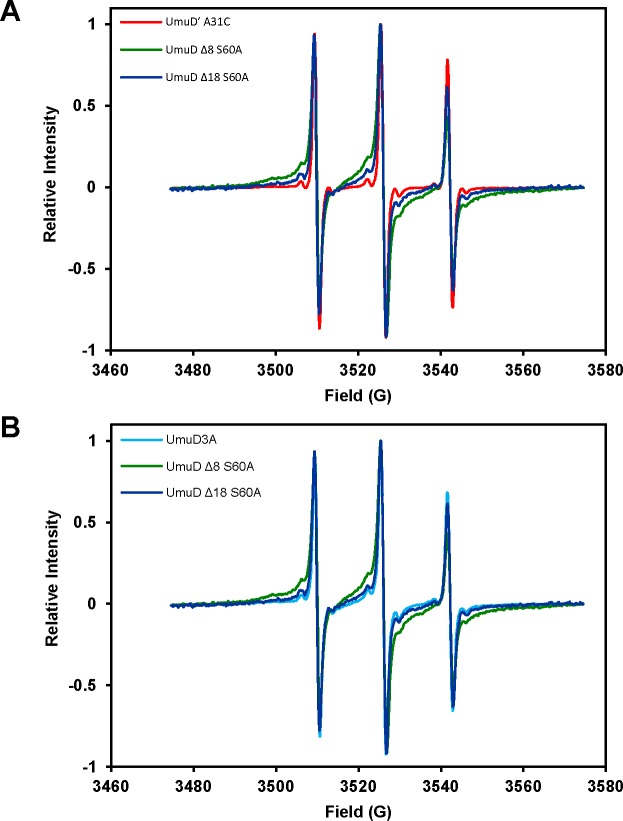
Continuous wave EPR spectra of UmuD variants. (**A**) Overlaid spectra of UmuD′ A31C and the truncation variants: The N-terminal arms of UmuD exhibit increased mobility when shortened. (**B**) Overlaid spectra of truncation variants with UmuD 3A demonstrate greater intermediate and slow-motional contributions to the spectral line shape of the truncation variants.

Site directed spin labeling (SDSL) allows for detection of conformational changes as well as local dynamics in a protein by electron paramagnetic resonance (EPR) spectroscopy. Three UmuD variants were modified with the paramagnetic spin label 3-iodomethyl-1-oxy-2,2,5,5-tetramethylpyrroline (iodomethyl spin label, IMSL) which specifically reacts with the sulfydryl group of cysteine residues. UmuD truncation variants UmuD 8 S60A and UmuD 18 S60A were labeled at the natural C24 position, and UmuD S60A C24A A7C was labeled at residue 7 near the end of the full-length arm. The spectra appear to be the superposition of spectra from at least two subpopulations of the nitroxide spin label: one displaying the characteristic three-line spectrum of a nitroxide undergoing fast motion, and additional components with broader lines indicating varying degrees of slower motion. Fig 1D compares spectra from the previously characterized UmuD S60A variant [10] with the truncated variants UmuD 8 and UmuD 18. The spectrum of UmuD S60A (purple) exhibits peaks from a relatively immobilized species (arrow, I) and from a more mobile population (arrow, II) in addition to the characteristic three-line spectrum. We previously demonstrated a temperature-dependent equilibrium between these two components [10]. When the arm is truncated (green spectrum, UmuD 8 S60A), the spectrum reflects an increase in the relative amount of the more mobile component, which becomes more pronounced as the truncation is increased in the UmuD 18 S60A construct (Fig 1D, blue). This is consistent with the conclusion that the arm becomes more mobile as it is shortened. UmuDʹ A31C, which was previously shown to exhibit only a fast-motional component [10], is compared with the two truncation variants of the present study in Fig 2A. This comparison clearly highlights the immobile component that is present upon partial truncation of the arms. As expected, the immobile component becomes more prominent as the length of the arm is increased. Similarly, comparison of spin-labeled UmuD 3A, which shows only intermediate and fast-motional components [10], with UmuD 8 S60A and UmuD 18 S60A reveals more intermediate and slow-motional components than for UmuD 3A (Fig 2B). We constructed UmuD A7C C24A S60A to place a spin label near the end of the N-terminal arm in the expectation that this would exhibit only a fast motional component, similar to UmuDʹ A31C; however, to our surprise this construct exhibits some less-mobile component, which could indicate some structure in the extreme N-terminal ends of the UmuD arms (S1 Fig).

### UmuD 8 cleaves efficiently, UmuD 18 is not cleavable

**Fig 3:**
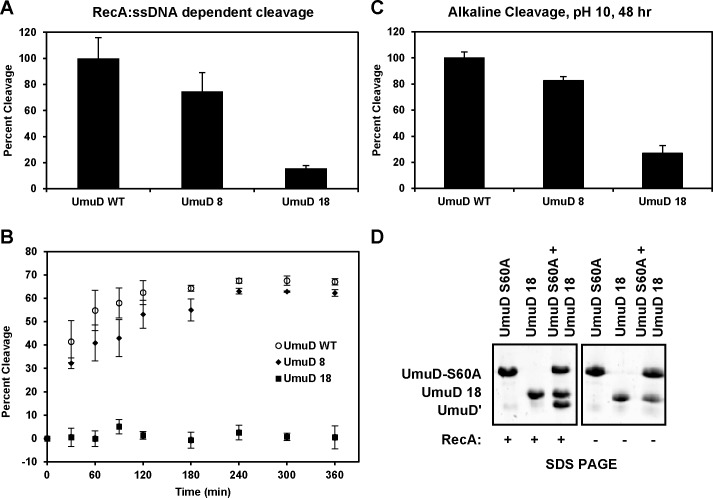
UmuD 8 cleaves as efficiently as wild-type UmuD; UmuD 18 does not cleave to UmuDʹ (**A**) Relative cleavage to UmuDʹ in the presence of RecA:ssDNA nucleoprotein filament. UmuD proteins at a concentration of 10 μM were used and the reaction was carried out for 1 h at 37°C. Percent cleavage product was determined as a ratio of the density of the UmuDʹ band to the total density of the uncleaved UmuD proteins and UmuDʹ protein for each reaction. (**B**) Comparison of the kinetics of cleavage of UmuD 8, UmuD 18, and wild-type UmuD. Reactions were carried out over 6 h. (**C**) Results for cleavage to UmuDʹ under alkaline conditions (pH 10) for 48 h are also represented. Results are normalized to cleavage of wild-type UmuD to form UmuDʹ. (**D**) Mixing equal amounts of UmuD 18 with the active site variant UmuD S60A results in cleavage. UmuD proteins at 10 μM were used and cleavage was carried out at 37°C for 1 h.

Cleavage of UmuD to UmuD′ is required for the activation of pol V (UmuD′_2_C) in translesion DNA synthesis [1]. The removal of the N-terminal 24 amino acids is facilitated by binding of UmuD to the RecA:ssDNA nucleoprotein filament, which positions the UmuD active site residues S60 and K97 in the correct orientation for the cleavage reaction [14]. Cleavage of UmuD 8 and UmuD 18 was assayed alongside full-length UmuD. Cleavage of UmuD 8 was nearly as efficient as that of wild-type UmuD, whereas that of UmuD 18 was dramatically reduced (Fig 3A and 3B). We also assessed cleavage of UmuD 8 under alkaline conditions (pH 10) in the absence of the RecA:ssDNA filament; at pH 10, cleavage is less efficient overall, but the active site serine can be activated as a nucleophile without the addition of RecA:ssDNA [5]. Under alkaline conditions, similar to our observations with RecA-facilitated cleavage, the cleavage of UmuD 8 was similar to that of full-length UmuD (Fig 3C). UmuD 18 does not undergo RecA:ssDNA-facilitated cleavage appreciably (Fig 3A and 3B). To determine whether UmuD 18 has a functional active site, we performed a RecA:ssDNA-dependent cleavage assay in which UmuD 18 and the active site variant UmuD S60A were mixed and allowed to form heterodimers. The N-terminal arms of UmuD S60A can then be cleaved in *trans* by the active site of UmuD 18 [11, 15]. We found that cleavage in the context of UmuD S60A/UmuD 18 heterodimers was indeed efficient (Fig 3D). This confirms that UmuD 18 and UmuD S60A can form heterodimers and that the active site of UmuD 18 is competent for cleavage, suggesting that the cleavage defect of UmuD 18 is due to decreased binding of its arm to its globular domain and presumably not due to a defect in its ability to interact with the RecA/ssDNA filament.

### UmuD arm length attenuates affinity for binding sites on DNA polymerase III α subunit

The *umuD* gene products interact with the DNA pol III α polymerase subunit at two locations: at the N-terminal domain between residues 1–280 and at the C-terminal region between residues 956–975 [21]. Since UmuD contains no tryptophans and α contains eight, we measured the intrinsic fluorescence of α in the presence of increasing amounts of UmuD to determine equilibrium dissociation constants *K*_d_ for the UmuD truncation variants binding to α. We probed the affinity of UmuD 8 S60A and UmuD 18 S60A for three forms of the α subunit: full-length α, α1–280, and α917–1160. Our observations indicate that UmuD 8 S60A exhibits a strong affinity for the α917–1160 C-terminal fragment (*K*_d_ = 0.2 ± 0.4 μM) which is similar to the equilibrium dissociation constant determined for UmuD S60A binding to the same α fragment (*K*_d_ = 0.7 ± 0.3 μM) [21]. On the other hand, UmuD 18 S60A displayed a weaker affinity for the C-terminal fragment (*K*_d_ = 3.6 ± 0.4 μM), and the calculated equilibrium dissociation constant closely resembles the values determined for UmuD′ and UmuD 3A (*K*_d_ = 3.8 ± 0.9 μM and 3.4 ± 1.0 μM, respectively) (Table 2 and S2 Fig) [21]. These observations further support the idea that UmuD 8 mimics UmuD whereas UmuD 18 is similar to UmuDʹ.

**Table 2:** Equilibrium dissociation constants^1^.

UmuD variants	Full-length α (μM)	α1–280 (μM)	α917–1160 (μM)
WT UmuD	1.1 ± 0.6	7.3 ± 1.4	13.9 ± 5.1
UmuD S60A	10.6 ± 2.9	10.3 ± 4.3	0.7 ± 0.3
UmuD′	10.9 ± 0.6	8.6 ± 1.0	3.8 ± 0.9
UmuD 8 S60A	8.1 ± 0.9	3.5 ± 0.8	0.2 ± 0.4
UmuD 18 S60A	12.2 ± 0.2	5.9 ± 0.9	3.6 ± 0.4
UmuD 3A	8.7 ± 1.5	9.3 ± 3.2	3.4 ± 1.0

^1^Equilibrium dissociation constants *K*_d_ (μM) for WT UmuD, UmuD S60A, UmuD′, and UmuD 3A were previously determined [21] and are reported here for ease of comparison.

**Fig 4:**
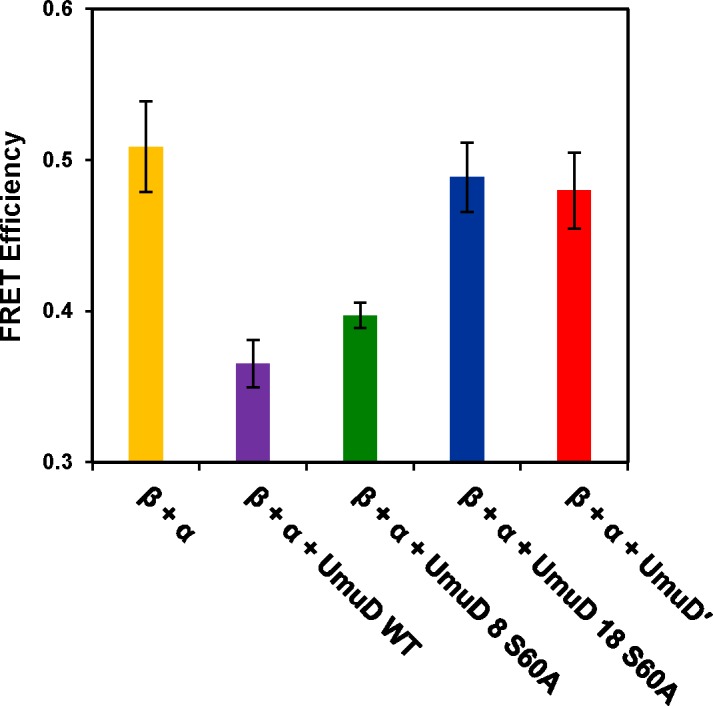
UmuD 8 S60A is able to disrupt the interaction between the α polymerase subunit and β processivity clamp while UmuD 18 S60A cannot. FRET was monitored between α labeled with Alexa Fluor 647 C_2_-maleimide and β labeled with Alexa Fluor 488 C_5_-maleimide. The bar graph shows FRET efficiency calculated in the presence and absence of purified UmuD proteins at 40 μM. Error bars represent standard deviation of three or more independent replicates.

Previous experiments have shown that full-length UmuD is capable of disrupting the interaction between the DNA pol III polymerase subunit α and the processivity clamp β [21]. When purified α and β subunits are labeled with acceptor and donor fluorophores, FRET is observed when donor-labeled β clamp is in the presence of acceptor-labeled α subunit. As expected and shown previously, FRET efficiency was significantly decreased in the presence of wild-type UmuD, but negligibly affected by the presence of UmuD′ (Fig 4) [21]. When the same experiment was performed using the UmuD arm truncation variants, we observed that UmuD 8 S60A was able to decrease FRET between α and β whereas in the presence of UmuD 18 S60A FRET efficiency was unchanged (Fig 4). This result is consistent with our observation that UmuD 8 S60A has a higher affinity for the C-terminal region of α similar to that of full-length UmuD and thus can compete for binding to the β clamp. Our observations show a correlation between longer N-terminal arm length, stronger affinity for the C-terminus of α, and the ability to disrupt the α-β complex.

### UmuD 8 is proficient for UV-induced mutagenesis; UmuD 8 and UmuD 18 do not confer resistance to UV radiation

**Fig 5:**
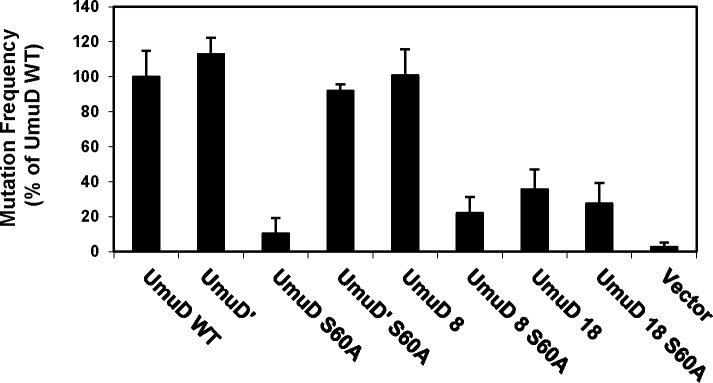
UmuD 8 is proficient for UV-induced mutagenesis. Mutagenesis assays were performed in strain GW8017.

**Fig 6:**
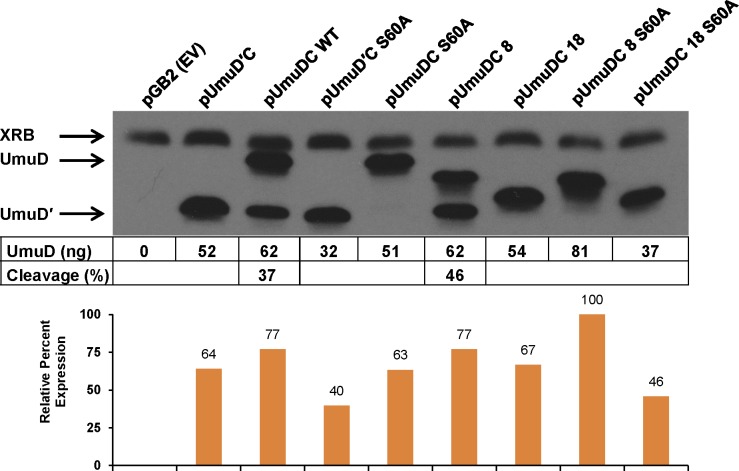
Steady state expression levels results of the UmuD proteins from plasmids in strain GW8017. Amount of UmuD in ng and percent cleavage of WT UmuD and UmuD 8 to UmuDʹ is also shown. A cross-reacting band (XRB) is indicated.

UmuD′_2_C performs TLS on UV-induced DNA damage and is required for UV-induced mutagenesis in *E*. *coli* [1]. As Pol V (UmuD′_2_C) inserts guanine opposite the 3′-thymine of (6–4) T-T photoproducts [37, 38], polymerase activity can be detected via the reversion of the *argE3* auxotrophic marker in the *E*. *coli* arginine biosynthetic pathway [33]. To determine the proficiency of UmuD 8 and UmuD 18 in UV-induced mutagenesis, we compared the mutation frequency of Δ*umuDC* strains harboring plasmid-borne full-length UmuD, UmuD′ and the truncated versions UmuD 8 and UmuD 18 (Fig 5). We also compared the corresponding active site variant, S60A, of each protein. We found that the mutation frequency of cells expressing UmuD 8 is similar to that of full-length UmuD, and as expected the non-cleavable UmuD 8 S60A shows greatly reduced UV-induced mutagenesis (Fig 5). The cleavage efficiency and expression level of UmuD 8 are also comparable to that of full-length UmuD *in vivo* (Fig 6). This suggests that UmuD 8 functions similarly to UmuD in this context, and is able to interact with protein partners that are required for UV-induced mutagenesis, including UmuC and RecA [1]. However, UmuD 18 shows reduced UV-induced mutagenesis (Fig 5). Although we did not detect cleavage of UmuD 18 *in vitro* or *in vivo* (Figs 3 and 6), cells expressing UmuD 18 had low but detectable mutagenesis. The greater UV-induced mutagenesis with this non-cleavable UmuD 18 truncation than other non-cleavable UmuD proteins can likely be attributed to the more dynamic N-terminal arms of UmuD 18, which would allow it to partially mimic UmuD′. In addition, the lower mutation frequency could be explained if accurate TLS is promoted by altered interactions of UmuD 18.

**Fig 7:**
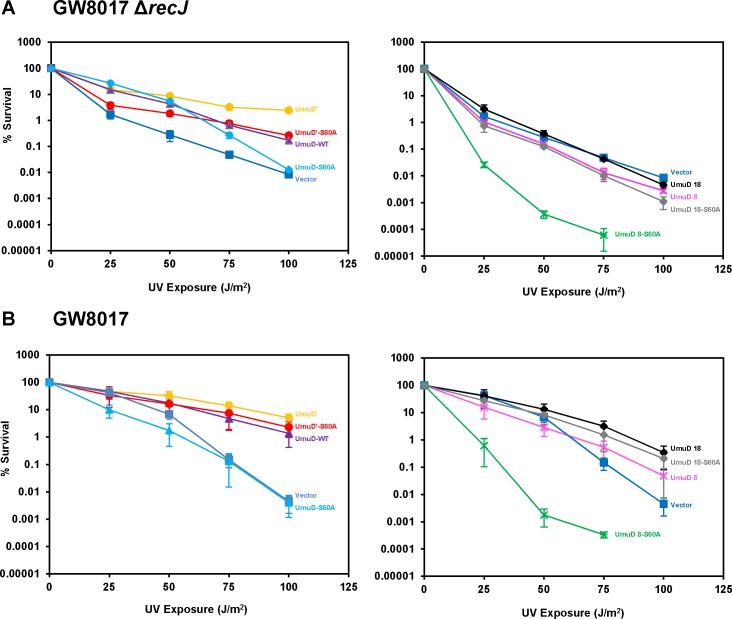
UmuD 8 S60A confers sensitivity to UV light. (**A**) UV Survival in strain GW8017 Δ*recJ* which lacks chromosomal *umuDC* and *recJ*. (**B**) UV survival in GW8017. Plasmids encode *umuDC* that vary only in the *umuD* construct.

**Fig 8:**
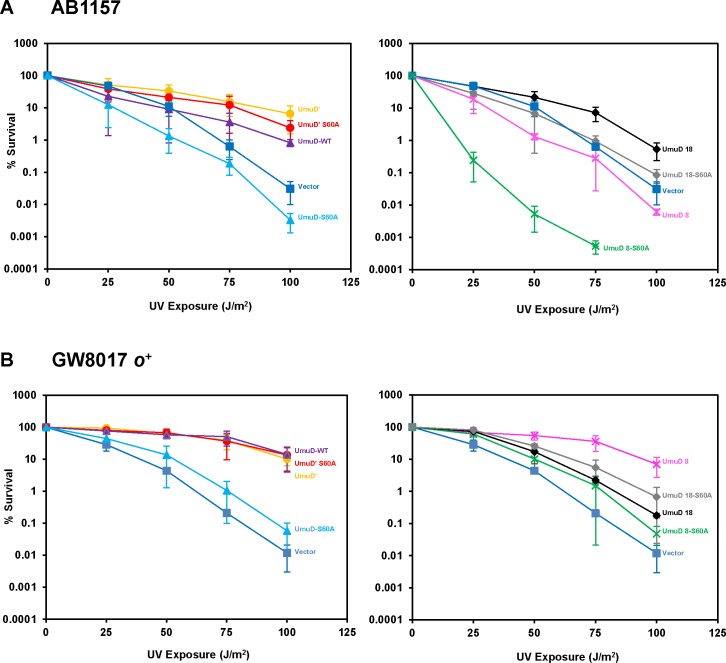
Sensitivity induced by UmuD 8 S60A is alleviated by tighter control over expression. (**A**) UV Survival in strain AB1157 which has chromosomal *umuDC*. UmuD 8 and UmuD 8 S60A confer UV sensitivity. (**B**) UV Survival of pUmuDC variants with wild-type promoter (*o*^+^) in GW8017.

**Fig 9:**
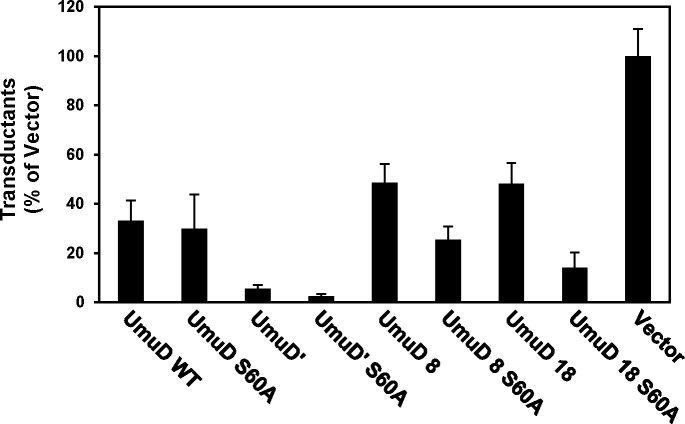
UmuD 8 and UmuD 18 do not inhibit homologous recombination to the same extent as UmuDʹC. UmuD proteins from plasmids pGY9739 (*umuDC*), pGY9738 (*umuDʹC*), and those harboring *umuD* variants UmuD 8 and UmuD 18 in strain GW8017 were expressed to determine the extent of inhibition of RecA-facilitated homologous recombination. Plasmids encode *umuDC* that vary only in the *umuD* construct.

It was previously reported that Δ*umuDC* Δ*recJ* strains are hypersensitive to UV radiation and that this phenotype can be suppressed by complementing with low-copy plasmids harboring the *umuDC* genes [16, 39]. RecJ is an exonuclease that aids in DNA replication restart by degrading DNA at stalled replication forks [39, 40]. In the absence of RecJ, replication restart is postponed and DNA synthesis is carried out by the TLS polymerase Pol V [39]. We found that cells expressing UmuD 8 or UmuD 18 display a similar level of sensitivity to UV light as cells harboring empty vector (Fig 7), and that UmuD 8 S60A surprisingly sensitized cells to UV, to an even greater extent than UmuD S60A. This extreme sensitivity to UV light conferred by UmuD 8 S60A was also observed for a strain with *recJ* (GW8017, Fig 7) and even in the context of AB1157 cells that harbor wild-type *umuD* on the chromosome (Fig 8). This phenotype was unexpected as UmuD 8 is proficient for UV-induced mutagenesis and presumably the UmuD 8 S60A variant could be cleaved by the chromosomally-encoded *umuD* to form UmuD′. We constructed corresponding plasmids expressing catalytically-deficient UmuC (D101N) or lacking *umuC* altogether and found that in both contexts, UmuD 8 S60A conferred UV hypersensitivity (S3 Fig). The *umuDC*-encoding plasmid we typically use for these experiments harbors a promoter mutation resulting in higher-than-normal expression levels [31, 41]. We therefore constructed an *o*^+^ version of the plasmid expressing UmuD 8 S60A and observed that this failed to confer UV hypersensitivity (Fig 8), thus indicating that the extreme sensitivity to UV light caused by UmuD 8 S60A is due to a copy-number effect. Our observation that cells harboring the *o*^C^_1_ version of UmuD 8 S60A are extremely sensitive to UV light suggests that elevated levels of UmuD 8 S60A can be harmful to cells. We next examined another characteristic phenotype of *umuDC*, specifically the inhibition of RecA-mediated homologous recombination by elevated levels of UmuDʹC [42–47]. UmuD 8 and UmuD 18 show similar levels of inhibition of RecA-mediated homologous recombination as full-length UmuD, again indicating that the truncated proteins appear to be proficient for interaction with RecA. UmuD 18 S60A inhibits RecA-mediated homologous recombination, suggesting it partially mimics UmuDʹ in its interactions with RecA-coated DNA (Fig 9).

## Discussion

The goal of this study was to characterize two truncation variants of the *E*. *coli* polymerase manager protein UmuD. We used several *in vivo* and *in vitro* techniques to investigate the effect of N-terminal arm length on protein conformation and activity. Surprisingly, we discovered that the variant UmuD 8 caused UV sensitivity in cells when expressed from a plasmid. We then attempted to characterize the cause of this sensitivity. Given that UmuD 8 is cleavable and viable for UV-induced mutagenesis, we were surprised to find that UmuD 8 was unable to confer resistance to UV in a Δ*umuDC* Δ*recJ* strain by complementation (Fig 7). Moreover, UV survival of a strain possessing a chromosomal copy of *umuDC* (AB1157) and harboring a low-copy plasmid bearing UmuD 8 was decreased relative to this strain harboring an empty vector (Fig 8). Cells are even more sensitive to UV when harboring a plasmid encoding the non-cleavable UmuD 8 S60A. The observed UV hypersensitivity phenotype was independent of UmuC catalytic activity in the context of Pol V, as introduction of the *umuC104* allele (D101N), which renders UmuC catalytically inactive [48], into the respective plasmid constructs did not confer resistance to UV. Complementation by plasmid-borne UmuD 18, on the other hand, promoted survival relative to empty vector in the case of both strains. This result was also surprising as UmuD 18 is non-cleavable and renders cells only weakly mutable (Figs 3 and 5). Plasmids used for complementation contain the *o*^*C*^_1_ point mutation in the *umuDC* operator, which decreases the regulation of plasmid-borne *umuDC* gene product expression by preventing LexA binding (S4 Fig) [31]. When reverted to the wild-type *o*^+^ operator sequence, the UV hypersensitivity phenotype observed for plasmid-borne UmuD 8 was alleviated. We therefore attribute this phenotype to a copy-number effect.

X-ray and NMR structures of UmuD′ [14, 49] show that the N-terminal arms are dissociated from the globular domain and are predominantly unstructured. While a high resolution structure of full-length UmuD has not been solved, homology models (Fig 1A) and observations from biophysical experiments indicate that the full-length N-terminal arms are dynamic, but also can stably bind to the globular domain [10, 11, 13, 50]. Indeed, interaction between arm residues Cys24 and Gly25 and globular domain active site residues Ser60/Lys97 is required for cleavage to occur. Both *in vivo* and *in vitro* cleavage experiments show that UmuD 8 cleaves as efficiently as wild-type UmuD whereas UmuD 18 is not cleavable (Figs 3 and 6). The single melting transition observed in thermofluor (Fig 1B) and elevated BMH cross-linking efficiency (39%, relative to 25% for WT UmuD and 41% for UmuD3A; Fig 1C) suggest that the conformation of UmuD 18 is more similar to that of UmuD′ in which the truncated arms weakly associate with the globular domain. The triple mutant UmuD 3A (T14A, L17A and F18A) possesses full-length arms, but is non-cleavable [10, 11, 13]. The three point mutations in UmuD 3A prevent interaction between the arms and globular domain. We propose that, like UmuD 3A, UmuD 18 is structurally similar to UmuD′, and its arms are not cleavable because they interact with the globular domain far more weakly than those of full-length UmuD.

The *umuD* gene products have been shown to interact with an increasingly large number of partner proteins. The UmuD interactome includes translesion DNA polymerases DinB and UmuC, RecA, subunits α, β, and ε of replicative DNA polymerase III, as well as proteases Lon and ClpXP [1, 5–7, 12, 21, 23, 27, 33, 51, 52]. Many of these interactions demonstrate preference for either UmuD or UmuD′. In addition, because UmuD variants lacking the N-terminal seven or eight residues maintain their interactions with both α (Table 2) and β [12], the disruption of α-β binding by UmuD 8 may be due to competitive interactions of UmuD 8 with both α and β. On the other hand, both UmuD 8 and UmuD 18 show reduced inhibition of RecA-mediated homologous recombination (Fig 9). Previous work identified several variants of UmuDʹ localized to the N-terminal arms that enhanced the inhibition of RecA-mediated recombination, specifically G25D, S28T, P29L, E35K, as well as T95R, suggesting an important role for the N-terminal arms region of UmuDʹ in modulating recombination [45]. In addition, UmuD single-cysteine derivatives that cross-linked most efficiently to RecA are at UmuD positions 34 and 81 [53]. These residues are present in both UmuD and UmuDʹ, and thus are also present in UmuD 8 and UmuD 18. Previous work showed that amino acid positions 19 and 24 are not implicated in interaction with RecA [53], so it is not unexpected that UmuD 8 and 18 inhibit homologous recombination to a similar extent.

Previous work from our lab has shown that UmuD interacts with two regions of α [21]. The first was localized to N-terminal residues 1–280 which make up the polymerase and histidinol phosphatase (PHP) domain [54–56], and the second was localized to the C-terminal region between residues 956–975. The C-terminal region of α binds more strongly to full-length UmuD-S60A relative to UmuD′ and UmuD 3A. UmuD′ and UmuD 3A share similar affinity for the α C-terminal region (*K*_d_ = 3.8 ± 0.9 and 3.4 ± 1.0 μM respectively), but differ in arm length. Considered together, these observations suggest that the interaction between the C-terminal region of α and UmuD requires that UmuD adopt an “arms-down” conformation in which the N-terminal arms of UmuD associate with its globular domain and create a specific binding site. UmuD 8 S60A exhibits similar affinity for the C-terminal region of α (*K*_d_ = 0.2 ± 0.4 μM) compared to UmuD S60A (*K*_d_ = 0.7 ± 0.3 μM [21]). It has previously been shown that UmuD S60A interacts with the α917–1160 fragment in an “arms-down” fashion [21]. Therefore, the N-terminal arms of UmuD 8 are also likely capable of associating with the C-terminal globular domains to achieve the preferred “arms-down” conformation. The binding constant determined for the interaction of UmuD 18 and α917–1160 (*K*_d_ = 3.6 ± 0.4 μM) mirrors the values calculated for UmuD 3A and UmuD′. Like the UmuD 3A and UmuD′ arms, the arms of UmuD 18 are also likely free in solution given that this variant is non-cleavable and exhibits relatively high levels of BMH cross-linking, and thus shows a weaker interaction with the C-terminal region of α.

In a previous study, UmuD arm variants similar to UmuD 8 and UmuD 18 were used to show that the β processivity clamp of DNA polymerase III has greater affinity for full-length UmuD and this affinity is somewhat reduced as residues are removed from the N-terminal arms, although β also binds to UmuD′ [12]. This preference was attributed to the presence of an interface created by the contact between the N-terminal arms and the C-terminal globular domain in full-length UmuD. The *umuD* gene products have been shown to inhibit DNA replication, which is presumably accomplished by their specific interactions with α, β, and likely other subunits of the replisome [3, 20, 21, 51, 57]. Indeed, in a FRET assay, energy transfer between fluorescently-labeled α and β subunits was decreased in the presence of wild-type UmuD, UmuD S60A, or UmuD 8, but no change in FRET was observed in the presence of UmuD′ or UmuD 18 [21]. Taken together, this and previous work support a model of specific interactions of the *umuD* gene products that dictate protein interactions important for regulating DNA replication.

## Supporting information

S1 Fig EPR spectrum of UmuD A7C C24A S60A shows reduced arm mobility. (PDF)

S2 Fig UmuD 8-S60A protein interacts with the Alpha Subunit of DNA Polymerase III at the N-terminal PHP domain. (PDF)

S3 Fig UV Sensitivity caused by UmuD 8 is not due to a deficient Pol V interaction. (PDF)

S4 Fig Wild-type SOS box sequence decreases UmuD steady-state expression and promotes cleavage. (PDF)
