# Supplementary material for: Altering the N-terminal arms of the polymerase manager protein UmuD modulates protein interactions
Source: PLoS One. 2017 Mar 8;12(3):e0173388. doi: 10.1371/journal.pone.0173388 (PMC5342242; doi:10.1371/journal.pone.0173388)
Supplement: S1 Fig — (PDF) [file pone.0173388.s001.pdf]

Altering the N-terminal arms of the polymerase manager protein UmuD modulates protein interactions

David A. Murison, Jaylene N. Ollivierre, Qiuying Huang, David E. Budil, and Penny J. Beuning

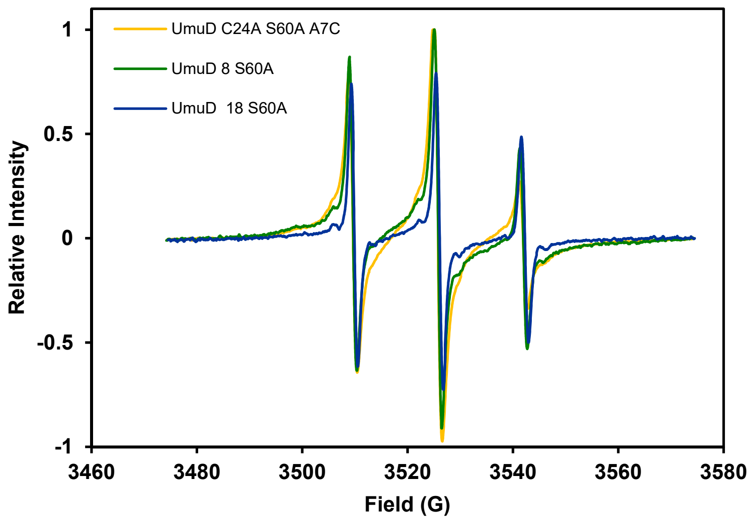

Supplemental Fig. 1. UmuD variant shows unexpected ordering at end of N-terminal arms. The variant UmuD C24A S60A A7C was created to represent maximum fast-motion line shape due to nitroxide labeling near the end of each N-terminal arm. Surprisingly, this variant exhibited a less-mobile line shape which may indicate some interaction between the extreme N-termini and globular domain.
