# Supplementary material for: Altering the N-terminal arms of the polymerase manager protein UmuD modulates protein interactions
Source: PLoS One. 2017 Mar 8;12(3):e0173388. doi: 10.1371/journal.pone.0173388 (PMC5342242; doi:10.1371/journal.pone.0173388)
Supplement: S2 Fig — (PDF) [file pone.0173388.s002.pdf]

Altering the N-terminal arms of the polymerase manager protein UmuD modulates protein interactions

David A. Murison, Jaylene N. Ollivierre, Qiuying Huang, David E. Budil, and Penny J. Beuning

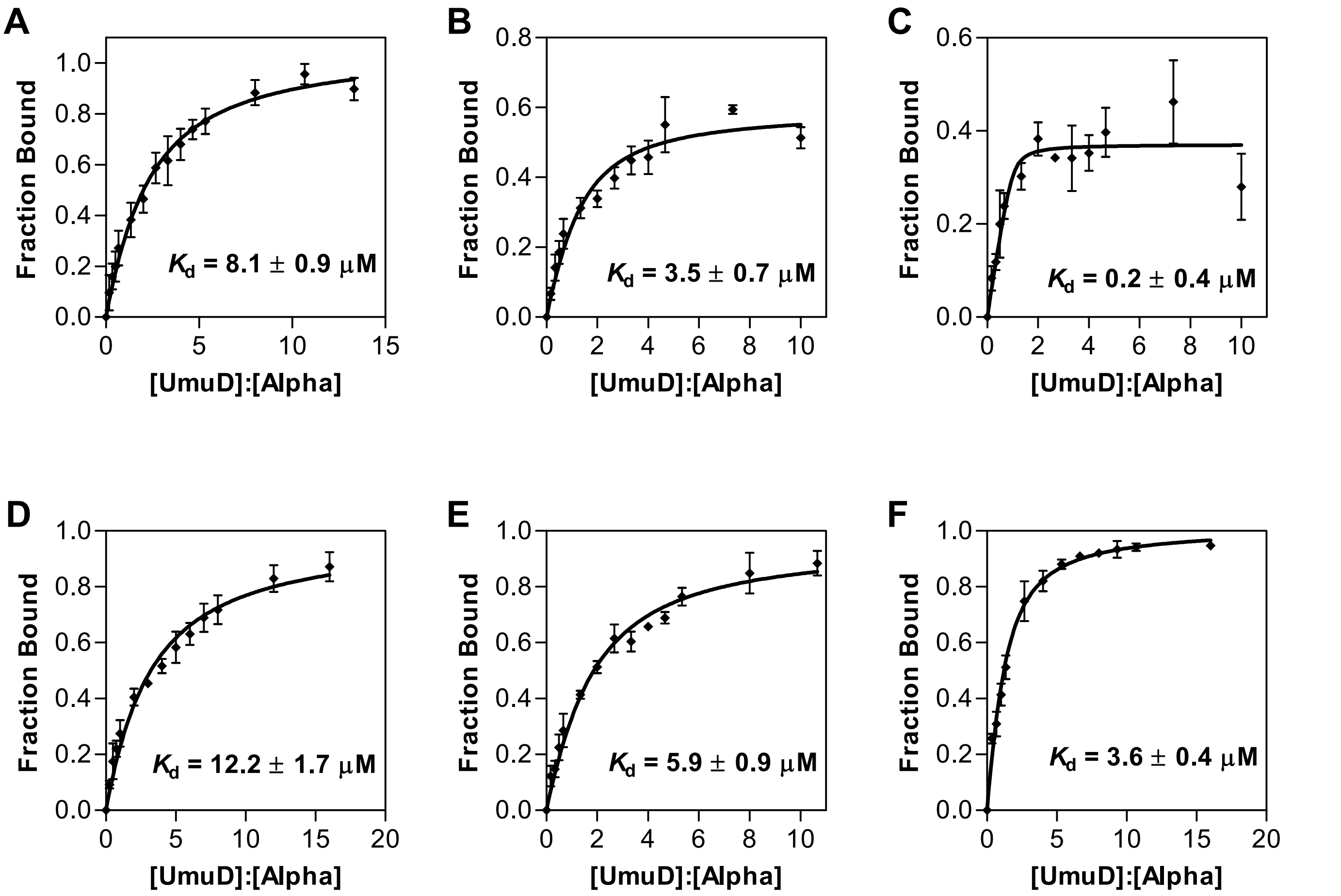

Supplemental Fig. 2. UmuD 8-S60A protein interacts with the Alpha Subunit of DNA Polymerase III at the N-terminal PHP domain. Binding constants were determined by tryptophan fluorescence for (A) UmuD 8 S60A and Alpha WT, (B) UmuD 8 S60A and Alpha1-280, (C) UmuD 8 S60A and Alpha917-1160, (D) UmuD 18 S60A and Alpha WT, (E) UmuD 18 S60A and Alpha1-280, and (F) UmuD18 S60A and Alpha917-1160. Curves represent the fraction of Alpha fluorescence quenched by increasing concentrations of UmuD protein which produced the K_d values shown.
