# Supplementary material for: Altering the N-terminal arms of the polymerase manager protein UmuD modulates protein interactions
Source: PLoS One. 2017 Mar 8;12(3):e0173388. doi: 10.1371/journal.pone.0173388 (PMC5342242; doi:10.1371/journal.pone.0173388)
Supplement: S3 Fig — (PDF) [file pone.0173388.s003.pdf]

Altering the N-terminal arms of the polymerase manager protein UmuD modulates protein interactions

David A. Murison, Jaylene N. Ollivierre, Qiuying Huang, David E. Budil, and Penny J. Beuning

A GW8017 *umuDΔC*

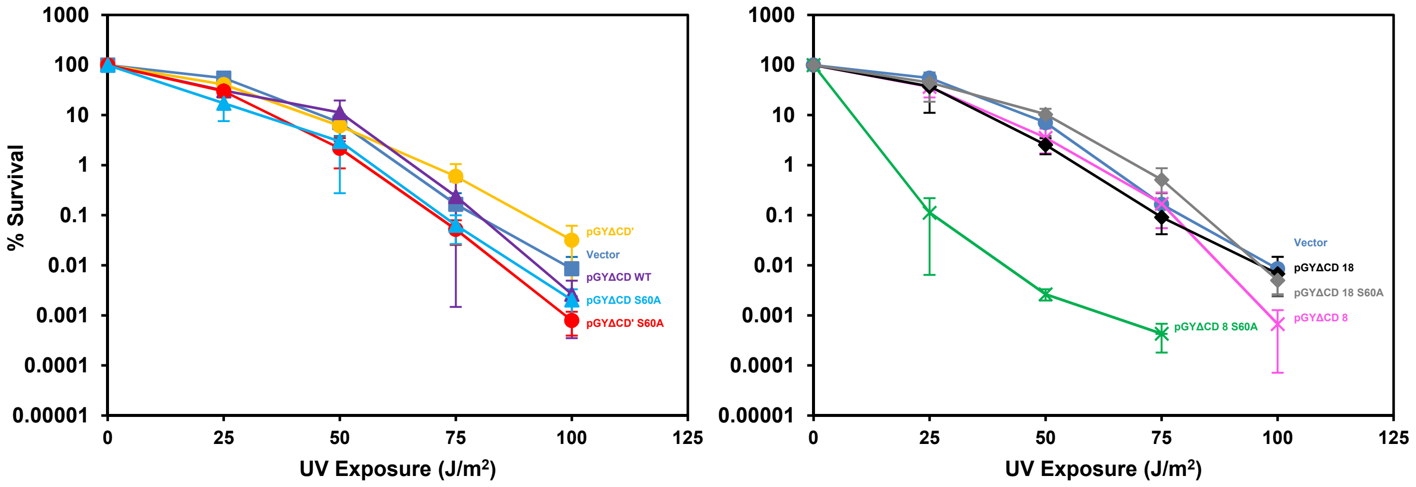

B GW8017 *umuC104*

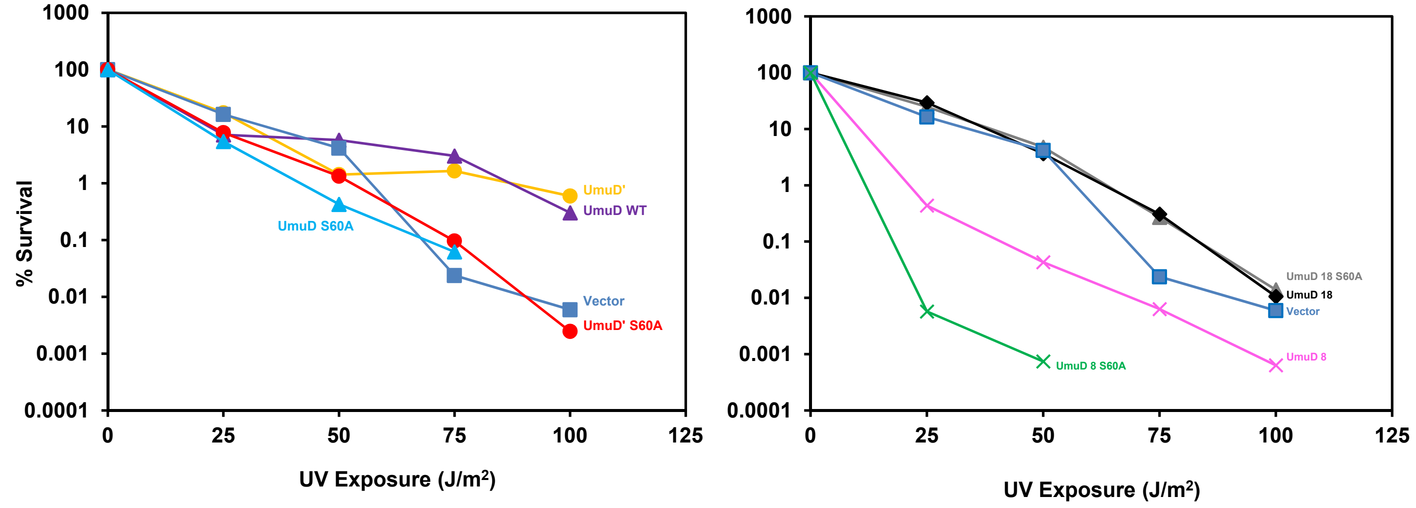

Supplemental Fig. 3. UV Sensitivity caused by UmuD 8 is not due to a deficient Pol V interaction. (A) UV Survival of plasmids possessing *umuD* only. The *umuC* sequence was removed entirely from *umuDC* operon in pGY9739 (B) UV survival of plasmids harboring *umuC104* allele (D101N) in GW8017. The point mutation D101N in UmuC inactivates the protein as a polymerase.
