# Supplementary material for: Altering the N-terminal arms of the polymerase manager protein UmuD modulates protein interactions
Source: PLoS One. 2017 Mar 8;12(3):e0173388. doi: 10.1371/journal.pone.0173388 (PMC5342242; doi:10.1371/journal.pone.0173388)
Supplement: S4 Fig — (PDF) [file pone.0173388.s004.pdf]

Altering the N-terminal arms of the polymerase manager protein UmuD modulates protein interactions

David A. Murison, Jaylene N. Ollivierre, Qiuying Huang, David E. Budil, and Penny J. Beuning

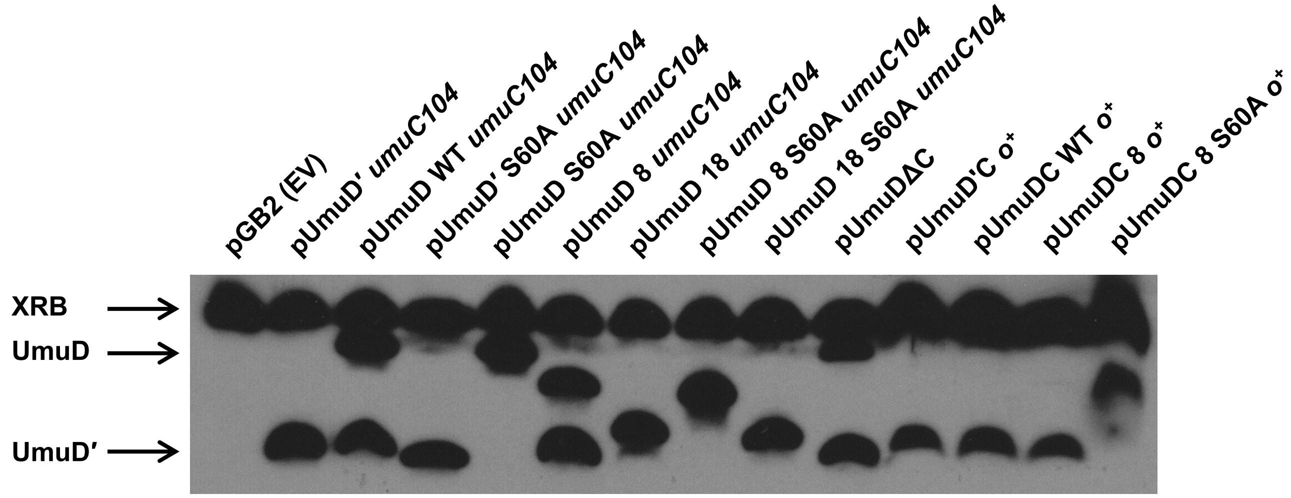

**UmuD (ng)**	**0**	**51**	**100**	**38**	**43**	**124**	**50**	**75**	**46**	**87**	**13**	**22**	**10**	**67**
**Cleavage (%)**			**47**			**54**				**58**		**100**	**100**	

Supplemental Fig. 4. Wild-type SOS box sequence decreases UmuD steady-state expression and promotes cleavage. Western blot of plasmids harboring either *umuC104* allele (D101N) and o^c_1 or wild-type *umuC* and o^+ in AB1157. The point mutation D101N in UmuC inactivates the protein as a polymerase. Amount of *umuD* gene products in ng and percent cleavage (where applicable) are shown. A cross-reacting band (XRB) is indicated.
